# Fulminant echovirus 11 hepatitis in male non-identical twins in northern Italy, April 2023

**DOI:** 10.2807/1560-7917.ES.2023.28.24.2300289

**Published:** 2023-06-15

**Authors:** Antonio Piralla, Alessandro Borghesi, Amelia Di Comite, Federica Giardina, Guglielmo Ferrari, Simona Zanette, Tiziana Angelica Figar, Micol Angelini, Camilla Pisoni, Antonino Maria Guglielmo Pitrolo, Stefania Paolucci, Francesca Rovida, Isabella Pellicioli, Ezio Bonanomi, Fausto Baldanti, Stefano Ghirardello

**Affiliations:** 1 Microbiology and Virology Department, Fondazione IRCCS Policlinico San Matteo, Pavia, Italy; 2 Neonatal Intensive Care Unit, Fondazione IRCCS Policlinico San Matteo, Pavia, Italy; 3 Department of Clinical, Surgical, Diagnostic and Paediatric Sciences, University of Pavia, Pavia, Italy; 4 Paediatric Intensive Care Unit, Ospedale Papa Giovanni XXIII, Bergamo, Italy; * These authors contributed equally to this work and share first authorship; ** These authors contributed equally to the work and share the last authorship

**Keywords:** enteroviruses, neonatal infection, hepatitis, echovirus 11 (E11)

## Abstract

Echovirus 11 (E11) has recently been associated with a series of nine neonatal cases of severe hepatitis in France. Here, we present severe hepatitis caused by E11 in a pair of twins. In one of the neonates, the clinical picture evolved to fulminant hepatitis. The E11 genome showed 99% nucleotide identity with E11 strains reported in the cases in France. Rapid genome characterisation using next generation sequencing is essential to identify new and more pathogenetic variants.

The World Health Organization (WHO) has recently reported an increasing number of severe neonatal infection associated with a specific species B enterovirus type, echovirus 11 (E11) [1]. By July 2022, nine cases of neonatal sepsis with liver disease and multi-organ failure had been reported in France from three metropolitan regions [2]. The European Centre for Disease Prevention and Control (ECDC) has recently included this virus in the ECDC Communicable Disease Threat Report [3]. According to the WHO, the public health risk for the general population is low, but the increase in reported cases remains a concern. Here, we report life-threatening E11 infection in a pair of dichorionic twins with a presentation and clinical course closely resembling those reported in the French cases [1,2].

## Description of cases and laboratory investigations

In April 2023, two non-identical, male, late-preterm twin brothers, P1 and P2, were transferred from the nursery to the neonatal intensive care unit (NICU) due to episodes of apnoea requiring respiratory support. They were later diagnosed with life-threatening E11 infection.

### Pregnancy history

The two infants were born to a healthy pregnant woman at 35 weeks and 3 days gestational age, following a spontaneous pregnancy. During pregnancy, their mother was given acetylsalicylic acid up to 35 weeks of gestation due to high risk of pre-eclampsia as assessed at the Bi-test, and enoxaparin sodium due to three previous spontaneous abortions. No other complications of the pregnancy were reported. All serological and biochemical laboratory tests during pregnancy were unremarkable. No episodes of diarrhoea were reported before partum by the mother or father. At 35 weeks and 1 day of gestation, the mother was admitted to the obstetrics unit for onset of labour with increased C-reactive protein (63 mg/L, norm: < 5.0), and betamethasone for the prophylaxis of neonatal respiratory distress and antibiotics were administered. A vaginal swab performed at admission later proved negative for group B streptococcus colonisation. One single fever episode with spontaneous resolution was registered at 35 weeks and 2 days of gestational age. The mother underwent a Caesarean section because of podalic presentation of P1 and progression of labour. Birth weights were 2,610 g (54th percentile) and 2,660 g (59th percentile), and Apgar scores 9 and 10 and 9 and 9 at 1 and 5 min for P1 and P2, respectively. No further virological investigations were performed on clinical specimens from the mother (i.e milk, plasma or stool).

### Neonate P1

On day 4 of life, P1 presented with episodes of apnoea and cyanosis and was transferred to the NICU on the following day for respiratory support with high flow nasal cannula (HFNC, maximum flow: 2 L/min). The infant underwent blood withdrawal for blood culture, later proven negative (5 days of monitoring), and was started on antibiotics. On day 6, a nasal swab for virological analyses, performed for the appearance of fever despite administration of broad-spectrum antibiotic, demonstrated the presence of enterovirus by specific real-time RT-PCR. Chest X-ray imaging on day 7 was normal. Antibiotics were stopped on day 7. Following clinical improvement, the respiratory support was stopped on day 10 of life, with further management of the infant in room air. 

Further virological testing on day 9 demonstrated the presence of enteroviral RNA in plasma (2.6 × 10^3^ RNA copies/mL) and urine (4.9 × 10^3^ RNA copies/mL). Laboratory testing was consistent with acute hepatitis with elevated liver enzymes and normal renal function, bilirubin levels and blood clotting values; the laboratory values at day 9 of life are reported in the Table. In the following days, the infant showed progressive clinical improvement with normalisation of liver enzymes on day 18 and was discharged home in good clinical condition on day 25 of life.

**Table:** Laboratory testing results on day 9 of life, twin neonates with echovirus 11 infection, Italy, April 2023

Laboratory test	P1	P2	Reference range
Aspartate transaminase (mU/mL)	294	2,740	11.0–39.0
Alanine transaminase (mU/mL)	291	11,925	11.0–34.0
Gamma-GT (mU/mL)	191	202	11.0–53.0
Creatinine (mg/dL)	0.39	0.46	0.30–0.70
Total bilirubin (mg/dL)	2.11	11.28	0.2–1.1
Direct bilirubin (mg/dL)	Not available	2.73	0.00–0.25
Plasma prothrombin (%)	94.00	Undetectable	70.00–120.00
Thromboplastin time (s)	42.90	Undetectable	20.00–32.00
Fibrinogen (mg/dL)	374	Undetectable	170–410
International normalised ratio	1.03	Unmeasurable	0.90–1.20
Hepatic cholinesterase (mU/mL)	5,656	2,986	5,300–12,900
Plasma ammonium (µg/dL)	160	192	19.0–94.0
Ferritin (ng/mL)	2,542	124,801	8–398

### Neonate P2

At the clinical disease onset, on day 6 of life, the infant presented with hypoxaemic respiratory distress requiring respiratory support on HFNC. On day 12, because his respiratory condition worsened and pleural effusion appeared, the infant received nasal continuous positive airway pressure with a maximum fraction of inspired oxygen (FiO2) of 0.25. The infant was weaned from any respiratory support on day 18. 

On day 9 of life, a blood count revealed thrombocytopenia (12,000/μl) with an extremely severe coagulopathy, undetectable clotting factors and fibrinogen, low haemoglobin (7.5 g/dL) and severely altered liver function; the laboratory values at days 9 of life are reported in the Table. EV RNA was detected in plasma (5.2 × 10^5^ RNA copies/mL) and urine (6.3 × 10^3^ RNA copies/mL). The infant underwent intensive daily transfusion treatment including 1–3 fresh frozen plasma and 1–2 platelet units per day, red blood cells (on days 10, 12 and 15) (transfusion volumes of 15–20 mL/kg), intravenous immunoglobulins (0.5 g/kg/day for 4 days) and albumin transfusion. Despite slow but progressive decrease in the liver enzymes and increase in cholinesterase levels, the infant remained transfusion-dependent until day 21. A first exchange transfusion due to high bilirubin levels (total bilirubin: 25.74 mg/dL; direct bilirubin: 12.35 mg/dL) was performed on day 17, and a second exchange transfusion due to persistently high bilirubin levels (total bilirubin: 26.7 mg/dL; direct bilirubin: 12.3 mg/dL) and elevated plasma ammonium (300 μg/dL) was performed on day 20 of life. Lumbar puncture was not performed due to the high risk of bleeding. The cerebral ultrasound on day 7 of life revealed enlarged lateral ventricles and two hyperechoic cerebellum lesions of haemorrhagic origin. No seizures were detected on electroencephalographic monitoring. 

On day 21, the infant was transferred to another paediatric intensive care unit for further management and possible consideration for liver transplantation. He was breathing spontaneously in room air and still transfusion-dependent. At the time of this report, the patient is still hospitalised, intubated in weaning from mechanical ventilation; thrombocytopenia persists without the need for platelet transfusion; the liver enzymes have normalised, but bilirubin concentration is still high (> 20 mg/dL, norm: 0.2–1.1) and due to coagulopathy, the infant requires plasma and cryoprecipitate transfusions at occurrence. Due to the persistence of severe hepatic insufficiency with ascites, the patient will be evaluated for liver transplantation.

## Phylogenetic and molecular analysis of the E11 strains

Enterovirus typing was performed in urine for P1 and in plasma samples for P2 by whole genome sequencing (WGS) and showed the presence of E11 strains. For WGS, we used the metagenomic approach as previously described by Kufner et al. [4]. Reads were mapped to the reference genome OQ927998 using the INSaFLU pipeline (https://insaflu.insa.pt) [5]. Phylogenetic analysis was performed on complete genome sequences obtained from urine of P1 (E11/ITA/01032786/2023) and plasma samples of P2 (E11/ITA/01032793/2023) (Figure). A maximum likelihood phylogenetic tree was inferred using the IQ-TREE web server (v1.6.8) [6], and the robustness of branches was evaluated using the Shimodaira–Hasegawa approximate likelihood-ratio test (SH-aLRT) and ultrafast bootstrap approximation tests. The Italian strains clustered with French strains collected in 2023 [2] (Figure), which together composed a divergent lineage. The average nucleotide identity based on the complete genome sequence was 99.3% (range: 98.9–99.6) between Italian and French E11 strains belonging to lineage 1, and it was 81.6% (range: 72.7–89.9) between the Italian strains and all other E11 genomes of good quality that were available and retrieved from GenBank (n = 93).

**Figure:**
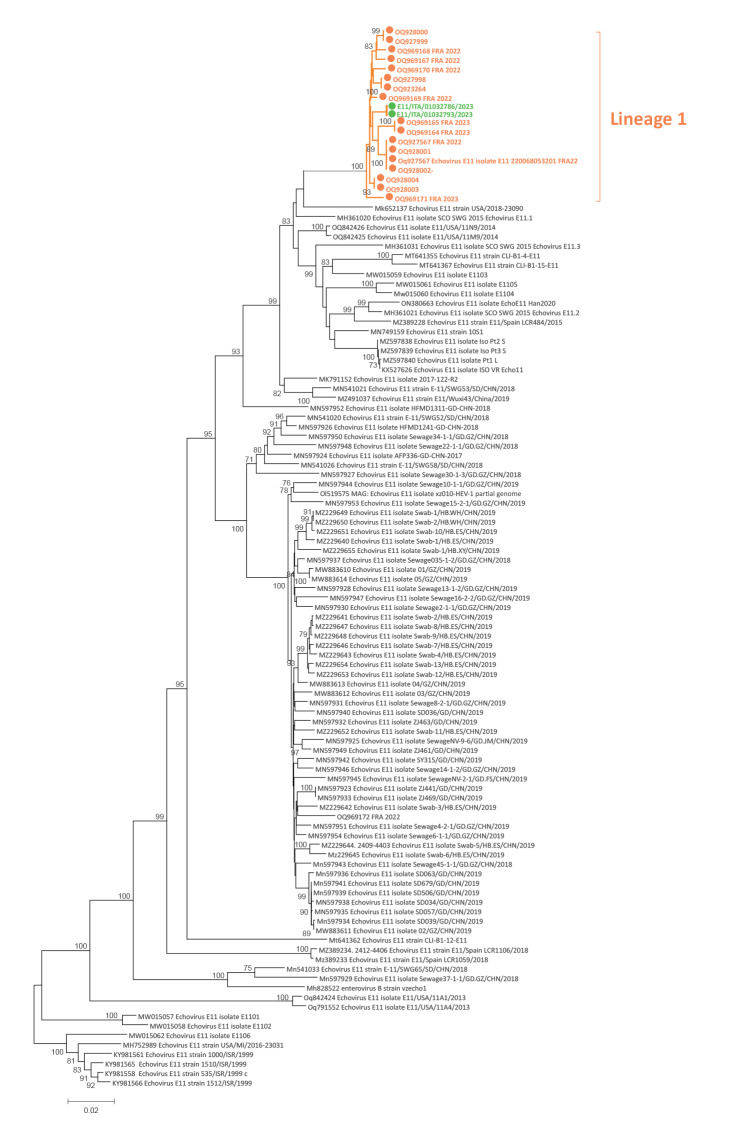
Phylogenetic tree of echovirus 11 complete genome sequences (n = 112)

Green: sequences from the two neonates reported in this manuscript; orange: sequences from neonatal cases reported in France [2].

## Discussion

An increase in the incidence and severity of acute and fulminant hepatitis associated with an emerging lineage of E11 in neonates, with marked prevalence in male twins, is currently observed in France [1,2]. The E11 and other non-polio enteroviruses have been circulating continuously in the European Union [7]. Several E11 lineages were circulating in the same geographical region at the same time [8]. A recombinant origin of the strain found in France was hypothesised by Grapin et al. [2], however, further analyses are needed to elucidate the origin of this divergent lineage as previously done for other E11 lineages [8].

Manifestations of neonatal E11 infections may range from asymptomatic to severe fatal multi-system disease [9]. In accordance with some other studies, severe E11 infection in our neonates was associated with a haemorrhage-hepatitis syndrome that causes hepatitis with liver dysfunction and coagulopathy as previously reported [2,10]. The clinical course of the twin brothers described here was consistent with that reported in the French cohort: fulminant, with sudden onset, quick worsening of the clinical conditions and liver function and development of life-threatening hepatitis, although with different severity and duration in the two infants. For both infants, survival was strictly dependent on rapid recognition of the infection and timely administration of intensive care. According to the French report and ours, a host genetic predisposition in male and twin categories might be hypothesised [2]. 

## Conclusion

The present report together with data of recent E11 cases in France direct public health attention to non-polio enteroviruses and their rare but severe clinical presentations. The risk factors for a severe course and the underlying causative mechanisms still need to be elucidated. The hospital-based enterovirus surveillance in France and Italy is voluntary, but these two reports highlight the need for active surveillance protocols in all cases with unexpected clinical presentations. In addition, complete genome sequencing could help with precise typing and molecular characterisation of emerging and re-emerging pathogenic variants, including identifying recombinant strains. 

## References

[1] World Health Organization (WHO). Enterovirus infection – France. Geneva: WHO; 2023Available from: https://www.who.int/emergencies/disease-outbreak-news/item/2023-DON469

[2] Grapin M Mirand A Pinquier D Basset A Bendavid M Bisseux M Severe and fatal neonatal infections linked to a new variant of echovirus 11, France, July 2022 to April 2023. Euro Surveill. 2023;28(22):2300253. 10.2807/1560-7917.ES.2023.28.22.2300253 37261730 PMC10236930

[3] European Centre for Disease Prevention and Control (ECDC). Weekly bulletin. Communicable Disease Threats Report. Week 18, 30 April–6 May 2023. Stockholm: ECDC; 2023. Available from: https://www.ecdc.europa.eu/en/publications-data/communicable-disease-threats-report-30-april-6-may-2023-week-18

[4] Kufner V Plate A Schmutz S Braun DL Günthard HF Capaul R Two years of viral metagenomics in a tertiary diagnostics unit: evaluation of the first 105 cases. Genes (Basel). 2019;10(9):661. 10.3390/genes10090661 31470675 PMC6770117

[5] Borges V Pinheiro M Pechirra P Guiomar R Gomes JP. INSaFLU: an automated open web-based bioinformatics suite "from-reads" for influenza whole-genome-sequencing-based surveillance. Genome Med. 2018;10(1):46. 10.1186/s13073-018-0555-0 29954441 PMC6027769

[6] Minh BQ Schmidt HA Chernomor O Schrempf D Woodhams MD von Haeseler A IQ-TREE 2: New models and efficient methods for phylogenetic inference in the genomic era. Mol Biol Evol. 2020;37(5):1530-4. 10.1093/molbev/msaa015 32011700 PMC7182206

[7] Bubba L Broberg EK Jasir A Simmonds P Harvala H Redlberger-Fritz M Circulation of non-polio enteroviruses in 24 EU and EEA countries between 2015 and 2017: a retrospective surveillance study. Lancet Infect Dis. 2020;20(3):350-61. 10.1016/S1473-3099(19)30566-3 31870905

[8] McWilliam Leitch EC Cabrerizo M Cardosa J Harvala H Ivanova OE Kroes AC Evolutionary dynamics and temporal/geographical correlates of recombination in the human enterovirus echovirus types 9, 11, and 30. J Virol. 2010;84(18):9292-300. 10.1128/JVI.00783-10 20610722 PMC2937653

[9] Chuang YY Huang YC. Enteroviral infection in neonates. J Microbiol Immunol Infect. 2019;52(6):851-7. 10.1016/j.jmii.2019.08.018 31607572

[10] Wang P Xu Y Liu M Li H Wang H Liu Y Risk factors and early markers for echovirus type 11 associated haemorrhage-hepatitis syndrome in neonates, a retrospective cohort study. Front Pediatr. 2023;11:1063558. 10.3389/fped.2023.1063558 37090924 PMC10117901

