# Addendum for Euro Surveill. 2023;28(24)

**DOI:** 10.2807/1560-7917.ES.2023.28.29.230720a

**Published:** 2023-07-20

**Authors:** Eurosurveillance editorial team

**Affiliations:** 1 European Centre for Disease Prevention and Control (ECDC), Stockholm, Sweden

In the article entitled ‘Fulminant echovirus 11 hepatitis in male non-identical twins in northern Italy, April 2023’ by Piralla et al., published on 15 June 2023, the Data availability statement was amended with the information that the viral genome sequences E11/ITA/01032786/2023 and E11/ITA/01032793/2023 have been deposited in GenBank under accession numbers OR266366 and OR266367. This addition was made on 17 July 2021.

